# Automated field‐in‐field whole brain radiotherapy planning

**DOI:** 10.1002/acm2.13819

**Published:** 2022-11-10

**Authors:** Kai Huang, Soleil Hernandez, Chenyang Wang, Callistus Nguyen, Tina Marie Briere, Carlos Cardenas, Laurence Court, Yao Xiao

**Affiliations:** ^1^ The University of Texas MD Anderson Cancer Center UTHealth Graduate School of Biomedical Sciences Houston Texas USA; ^2^ Department of Radiation Physics The University of Texas MD Anderson Cancer Center Houston Texas USA; ^3^ Department of Radiation Oncology The University of Texas MD Anderson Cancer Center Houston Texas USA; ^4^ Department of Radiation Oncology The University of Alabama at Birmingham Birmingham Alabama USA

**Keywords:** automation, deep learning, field‐in‐field, whole‐brain radiotherapy

## Abstract

**Purpose:**

We developed and tested an automatic field‐in‐field (FIF) solution for whole‐brain radiotherapy (WBRT) planning that creates a homogeneous dose distribution by minimizing hotspots, resulting in clinically acceptable plans.

**Methods:**

A configurable auto‐planning algorithm was developed to automatically generate FIF WBRT plans independent of the treatment planning system. Configurable parameters include the definition of hotspots, target volume, maximum number of subfields, and minimum number of monitor units per field. This algorithm iteratively identifies a hotspot, creates two opposing subfields, calculates the dose, and optimizes the beam weight based on user‐configured constraints of dose‐volume histogram coverage and least‐squared cost functions. The algorithm was retrospectively tested on 17 whole‐brain patients. First, an in‐house landmark‐based automated beam aperture technique was used to generate the treatment fields and initial plans. Second, the FIF algorithm was employed to optimize the plans using physician‐defined goals of 99.9% of the brain volume receiving 100% of the prescription dose (30 Gy in 10 fractions) and a target hotspot definition of 107% of the prescription dose. The final auto‐optimized plans were assessed for clinical acceptability by an experienced radiation oncologist using a five‐point scale.

**Results:**

The FIF algorithm reduced the mean (± SD) plan hotspot percentage dose from 35.0 Gy (116.6%) ± 0.6 Gy (2.0%) to 32.6 Gy (108.8%) ± 0.4 Gy (1.2%). Also, it decreased the mean (± SD) hotspot V107% [cm^3^] from 959 ± 498 cm^3^ to 145 ± 224 cm^3^. On average, plans were produced in 16 min without any user intervention. Furthermore, 76.5% of the auto‐plans were clinically acceptable (needing no or minor stylistic edits), and all of them were clinically acceptable after minor clinically necessary edits.

**Conclusions:**

This algorithm successfully produced high‐quality WBRT plans and can improve treatment planning efficiency when incorporated into an automatic planning workflow.

## 1 INTRODUCTION

Brain metastases are the most common intracranial tumors.^1^ More than 9% of patients with cancer develop brain metastases, contributing to 308,102 new cases of brain cancers globally each year.^2^, ^3^ Management of brain metastases is usually palliative and can include chemotherapy, surgery, radiotherapy, or a combination of them. Reports show approximately 25%–50% of radiotherapy treatments worldwide are delivered with palliative intent.^4^, ^5^ Common radiotherapy techniques for brain metastases include stereotactic radiosurgery and whole‐brain radiotherapy (WBRT). The choice of technique depends on the number and sizes of the lesions as well as the tumor resectability and whether the patient is symptomatic.^6^ Although many well‐resourced clinics are moving toward supplementing WBRT with stereotactic radiosurgery to achieve delivery of more conformal doses to metastases while sparing healthy tissue, WBRT alone still plays a critical role in the management of brain metastases.^7^, ^8^, ^9^ Moreover, most patients present with advanced stages of disease, which further drives the need for palliative radiotherapy.

WBRT typically uses two parallel opposed lateral beams to cover the brain volume. The typical fractionation pattern used in both resource‐constrained and well‐resourced clinics is 30 Gy in 10 fractions.^4^, ^10^ To design the shape of such beams, organs at risk (OAR) are viewed in the beam's‐eye‐view, and clinicians manually design the shape of the beams to optimize coverage to the brain and cribriform plate while considering the dose to normal tissues, including the eyes and lenses. This process can be iterative to meet specified dose criteria for these structures. The field shape varies depending on clinical practices and preferences. Different clinical practices may prioritize different criteria, such as the cribriform plate coverage, dose constraint to the lenses, and brain dose coverage. Researchers have automatically generated customizable field apertures to satisfy different clinical requirements.^11^, ^12^ In the present study, we used a previously validated field generation model to generate field shapes for planning.^12^

Patients undergoing WBRT may experience radiation‐induced toxicities including acute, early‐delayed, and late adverse effects.^10^ Acute toxicities include fatigue, alopecia, dermatitis, nausea, and vomiting. Early‐delayed toxicities include neurocognitive deficits and somnolence. Acute and early‐delayed toxicities tend to self‐resolve within a matter of weeks. However, late toxicities do not self‐resolve and may have severe consequences, such as neurocognitive degeneration and radiation necrosis.^10^ Furthermore, because of the shape and anatomy of the human head, WBRT plans are susceptible to inhomogeneous dose distributions. Hotspots usually appear on the skull and at the back of the neck. Excessive hotspots can cause unnecessary radiation exposure to patients and should be minimized as much as possible.

The field‐in‐field (FIF) technique is often used in WBRT to reduce hotspots in plans because of its ability to operate in two dimensions. In this technique, subfields blocking the locations of hotspots are iteratively added to the plans to minimize hotspots while maintaining the desired target volume coverages. However, the process of manually adding subfields to plans and adjusting beam weights accordingly is time‐consuming and requires planner expertise. Therefore, resource‐constrained clinics may opt out of the FIF planning process.

Several investigators have worked to expedite WBRT planning via automation.^11^, ^13^ Although automatic FIF solutions exist for treatment sites other than the brain, such as the breast and rectum, such solutions have yet to be applied to WBRT. Current WBRT automation techniques are limited to field generation and dose calculation and do not include FIF optimization. A unique challenge in FIF automation with WBRT is that hotspots may appear in bone and become hollow and widely scattered as a result.^14^, ^15^ Such scattered hotspot shapes make it difficult for automatic solutions to choose which region to block while maintaining target coverage without introducing cold spots. In this study, we demonstrated that the automated FIF technique can be consistently and efficiently used to produce clinically acceptable WBRT plans.

## 2 METHODS

An automated FIF algorithm was developed and carefully configured for various planning variables defined via physician preferences. Performance of the FIF algorithm was quantitatively and qualitatively reviewed using dose metrics and physician review, respectively.

### 2.1 Data set

WBRT plans and the associated computed tomography (CT) scans for 17 patients who received treatment in our clinic from 2012 to 2018 were collected. The median (range of) number of slices, slice thickness, and pixel spacing for the CT scans were 123 (97–293), 3 mm (1–3 mm), and 1.03 mm (0.97–1.03 mm), respectively. All CT scans used 120 kVp.

**FIGURE 1:**
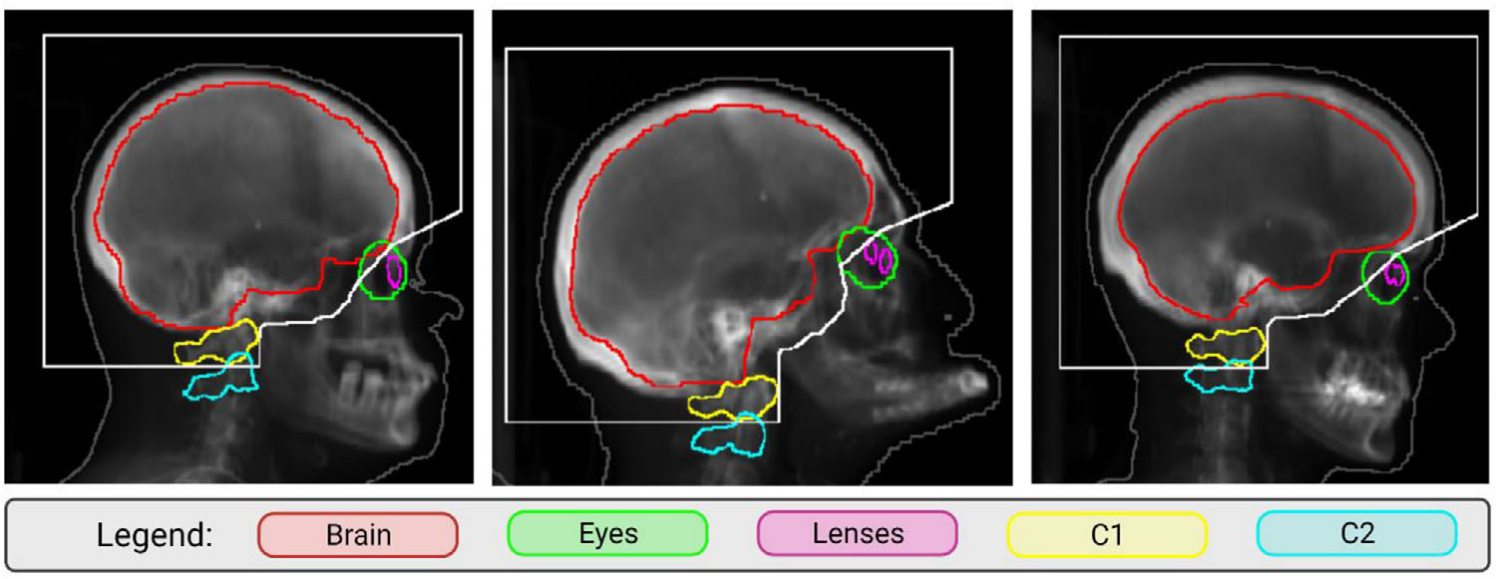
Examples of landmark‐based apertures (outlined in white) on digitally reconstructed radiographs for three patients

Each clinical plan was generated using a customizable, landmark‐based, and automated aperture generation algorithm.^12^ In this algorithm, the regions of interest are the brain, left eye, right eye, left lens, right lens, C1 vertebra, and C2 vertebra, which are automatically delineated using deep learning‐based methods.^16^, ^17^ Next, the regions of interest are projected into the beam's‐eye‐view. A field aperture is automatically generated using a landmark‐based approach. Jaws and multi‐leaf collimators (MLC) are then conformed to block the field to the aperture shape. Examples of automatically generated apertures are shown in Figure 1. Initial plans were then generated and initialized with equal beam weighting for the opposed beams to use in FIF optimization.

### 2.2 FIF algorithm design

**FIGURE 2:**
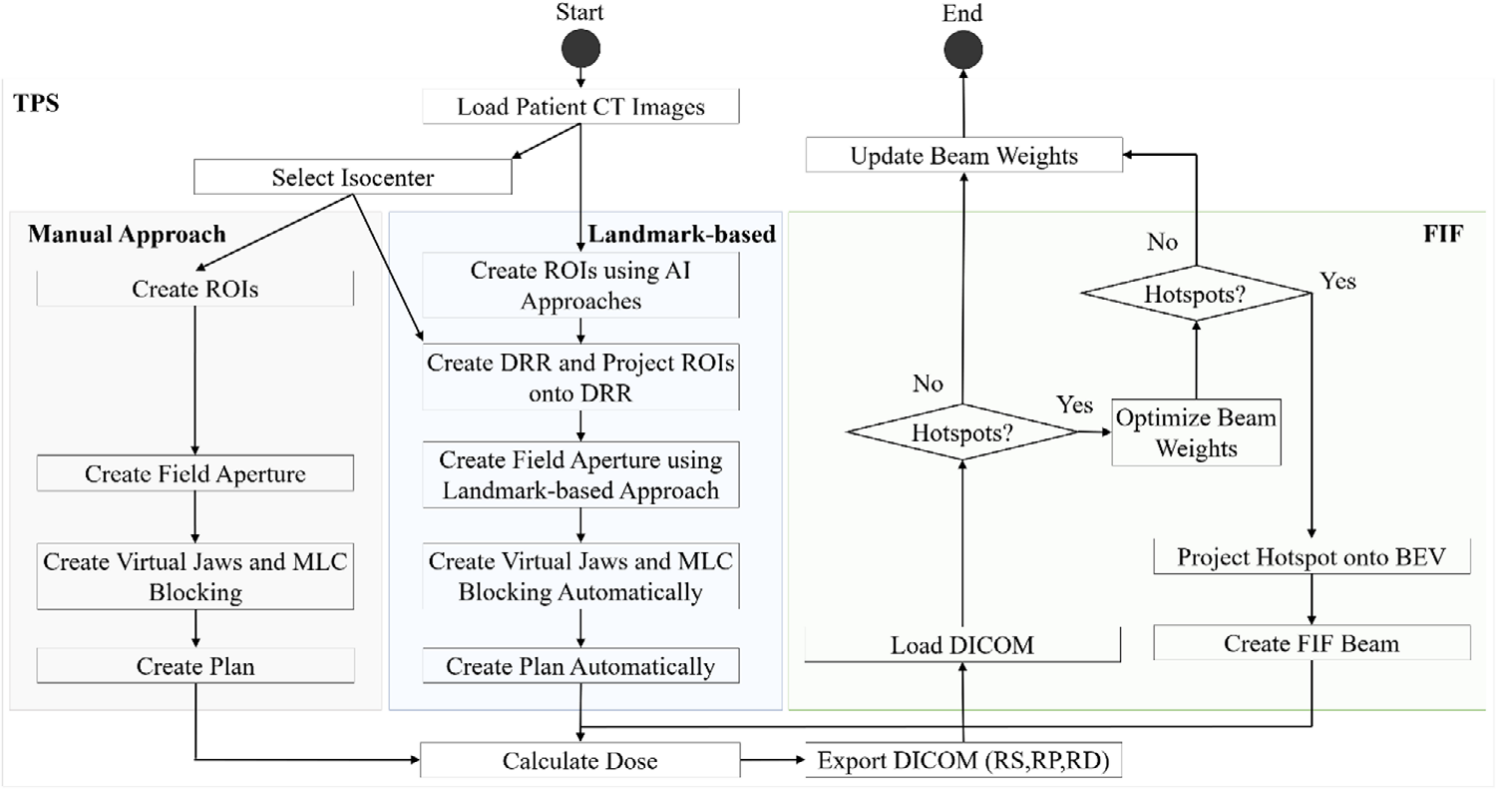
The workflow for the end‐to‐end field‐in‐field (FIF) automation process from predicting apertures to adding a plan with FIF compared with the manual approach to plan. ROIs, regions of interest; MLC, multileaf collimator; AI, artificial intelligence; DRR, digitally reconstructed radiograph; DICOM, Digital Imaging and Communications in Medicine; RS, radiotherapy structure file; RP, radiotherapy planning file; RD, radiotherapy dose file; BEV, beam's‐eye‐view

The FIF algorithm mimics the forward‐planning approach in clinics to reduce dose hotspots. All of the experiments in the present study were performed using RayStation treatment planning system (version 10B). The FIF algorithm was implemented and validated for rectal cancer treatment in both RayStation and Eclipse (version 15.5) treatment planning systems.^18^ Figure 2 shows the specific workflow for the end‐to‐end automation process, including the previously described FIF algorithm.^18^ The algorithm iteratively adds subfields to plans by locating the hotspots to block while performing beam weight optimization to minimize hotspots. The algorithm used the L‐BFGS‐B solver for beam weight optimization,^19^ which was selected based on our preliminary experience.

The following settings were determined according to our clinical practice. Hotspots were defined as any disconnected volume larger than 1 cm^3^ receiving 107% of the prescription dose (Rx). The maximum number of subfields that could be added to the plans was six. The energy of each beam was set at 6 MV. The prescribed dose for the brain volume was 30 Gy in 10 fractions. The normalization of the plans was set to 99.9% of the brain volume receiving 100% of the Rx. Also, the minimum number of monitor units per field was set to five. The FIF algorithm was further configured to add opposed lateral subfields iteratively and in parallel. Therefore, all the plans had an even number of total fields. Dose calculation was performed in the treatment planning system via scripting interface. A fixed decrement of 3% was chosen for thresholding hotspots at each iteration. This was selected as a compromise between causing cold spots if the decrement was too large and increasing the number of actual iterations if the decrement was too small. This meant that at the start of an iteration, if the plan had a maximum hotspot of 116% of the Rx, the hotspot of 113% of the Rx would be blocked, and so on. Based on the locations and the shapes of the iso‐dose lines, the MLC shapes of the sub‐fields were determined. At the start of every iteration, the maximum hotspot percentage was recalculated. The FIF algorithm stopped when the target dose level was reached (i.e., maximum hotspot percentage = 107% of the Rx), when six subfields were added to the plans, or when adding subfields would increase the size of the hotspot. All of these settings can be configured and modified for other clinical practices.

### 2.3 FIF algorithm testing

The FIF algorithm was tested using the automatically generated WBRT plans for the 17 patients. As described above, the algorithm's performance was quantitatively and qualitatively assessed using dose metrics and physician review, respectively. The average plan generation time was also quantified.

#### 2.3.1 Quantitative algorithm evaluation

To quantitatively assess the FIF algorithm's performance, various dose metrics were calculated for the automated FIF plans and compared against the clinically delivered plans. The hotspot dose percentage was quantified before and after running the FIF algorithm. Also, the maximum plan dose was reported. Coverage to the brain was quantified using the D99% (Gy), D95% (Gy), and D1% (Gy). The mean and maximum doses were calculated for the eyes and lenses, respectively. Additionally, planning parameters such as the total number of field segments and automated planning time were quantified. Other essential planning parameters were configured as described in the FIF algorithm design section and include the specific plan to load, prescriptions, maximum allowed iteration, subfield minimum and maximum MU, target dose percentage, whether to add wedges or to normalize by volume or point, and the optimization solver. All the reported quantitative values were based on the results after renormalization for plans initially scored as unacceptable but improved after renormalization during physician reviews. If the plans’ clinical acceptability did not improve after renormalization, the reported values were based on the original renormalization.

#### 2.3.2 Qualitative algorithm evaluation

To qualitatively assess the FIF algorithm's performance, physician review of the automatically generated FIF plans was performed. The physician scored each plan on a scale of 1–5: 5 = use as is, 4 = use with unnecessary minor edits, 3 = use with necessary minor edits, 2 = use with major edits, and 1 = do not use (Table 1). Plans with scores of 4 or 5 were deemed clinically acceptable. If a plan scored at or below a 3, the plan was renormalized and rescored to determine whether its clinical acceptability improved.

**TABLE 1:** The five‐point scale used for evaluating the quality of the whole‐brain radiotherapy (WBRT) plans

Score	Acceptability	Description
5	Acceptable: use as is	Clinically acceptable, could be used for treatment without changes
4	Acceptable: minor edits that are not necessary	Stylistic differences, but not clinically important; the current contours/plans are acceptable
3	Unacceptable: minor edits that are necessary	The edits are clinically important, but editing the automatically generated contours/plans is more efficient than starting from scratch
2	Unacceptable: major edits	Edits that are required to ensure appropriate treatment and are significant enough that the user would prefer to start from scratch
1	Unacceptable: unusable	Automatically generated contours/plans are so poor that they are unusable (e.g., wrong body area, outside confines of body)

## 3 RESULTS

### 3.1 Quantitative algorithm evaluation

**FIGURE 3:**
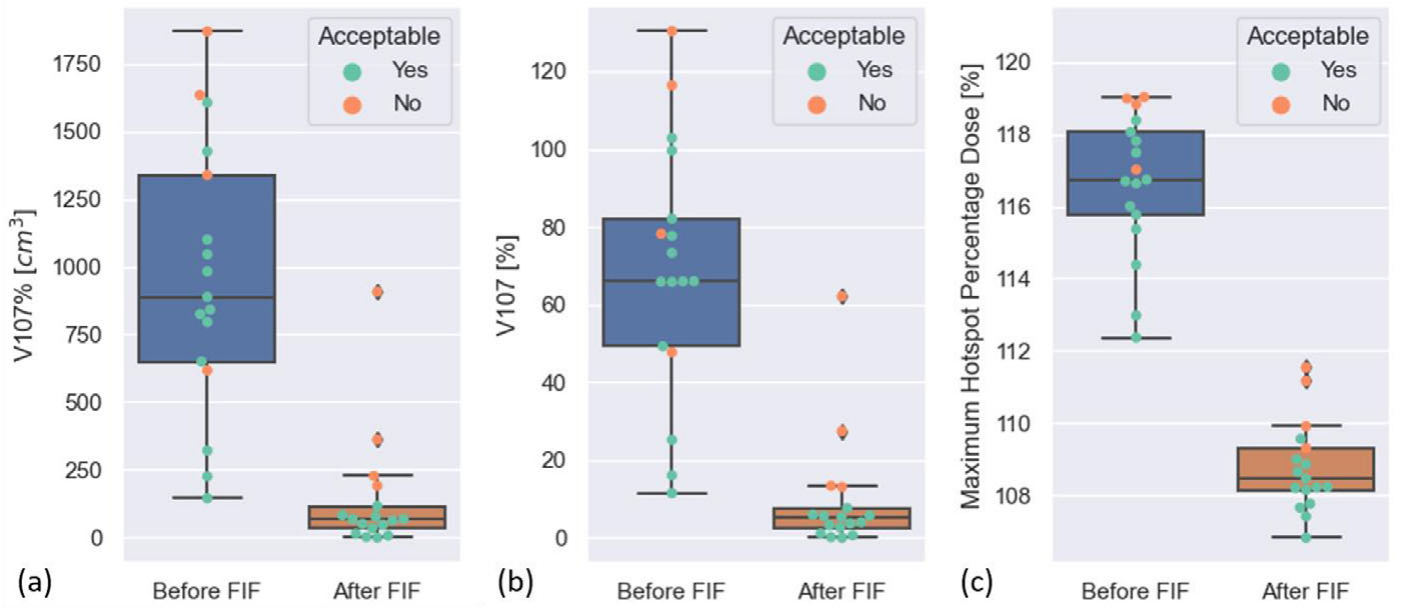
Box plots of the dose metrics used to quantitatively assess the field‐in‐field (FIF) algorithm's performance before and after applying the algorithm to the 17 whole‐brain radiotherapy (WBRT) plans. (a) The V107% (cm^3^). (b) The V107% (%) as a percentage of the brain structure. (c) The maximum hotspot percentage dose. The color of each data point indicates the clinical acceptability of the plans

As shown in Figure 3, we quantified the hotspots in V107% [cm^3^], V107% [%] as a percentage of the brain structure, and maximum hotspot percentage dose before and after FIF optimization. FIF optimization decreased the mean (± SD) plan hotspot percentage dose from 35.0 Gy (116.6%) ± 0.6 Gy (2.0%) to 32.6 Gy (108.8%) ± 0.4 Gy (1.2%). FIF optimization decreased the mean (± SD) hotspot V107% [cm^3^], from 959 ± 498 cm^3^ to 145 ± 224 cm^3^ for the plans without FIF optimization. Also, FIF optimization decreased the median hotspot volume V107% [cm^3^] from 888 cm^3^ to 68 cm^3^ for the plans without FIF optimization. The average V107% [%] as a percentage of the brain structure decreased from 69.0% to 10%. The maximum hotspot as a percentage of the Rx quantified for automatically generated plans before and after FIF optimization is also shown in Figure 3. The hotspots after FIF optimization had a lower dose coverage than did the hotspots before FIF optimization.

**FIGURE 4:**
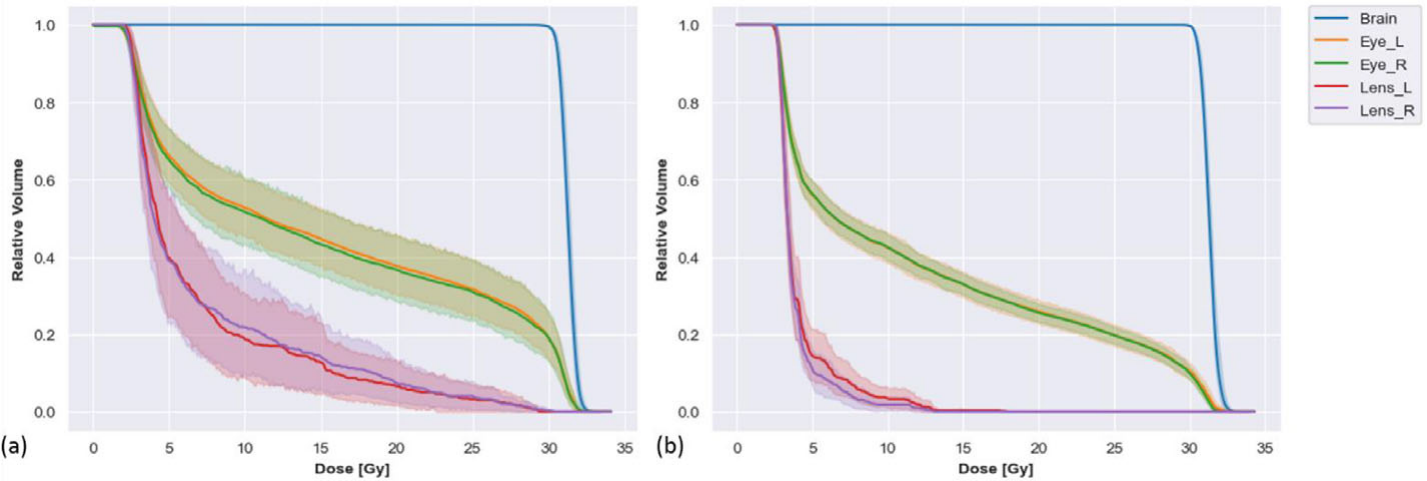
The distribution of the dose‐volume histogram (DVH) for different organs at risk (OARs) (a) for all 17 original plans and (b) for all 17 automatic field‐in‐field (FIF) plans

As shown in Table 2, we quantified dose coverage metrics for the brain, the mean dose to the left and right eyes, and the maximum dose to the left and right lenses. The mean (± SD) doses to the left and right eyes were 11.48 ± 2.03 Gy and 11.44 ± 1.68 Gy, respectively. The mean (± SD) maximum doses to the left and right lenses were 8.19 ± 4.15 Gy and 7.72 ± 2.96 Gy, respectively. The mean (± SD) D99, D95, and D1 to the brain were 30.33 ± 0.17 Gy, 30.63 ± 0.25 Gy, and 32.21 ± 0.29 Gy, respectively. Compared with the original plans, the FIF optimization on average decreased the mean dose to the left and right eyes by 3.47 Gy and 3.22 Gy, respectively. The FIF optimization on average decreased the maximum dose to the left and right lenses by 6.65 Gy and 8.05 Gy, respectively, compared with the original plans. The differences between the mean D99, D95, and D1 to the brain between the original and automated FIF plans were under 0.2 Gy. Figure 4 shows the distribution of dose‐volume histogram for the brain, left and right eyes, and left and right lenses across the 17 patients from the original plans and the automated FIF plans. The FIF algorithm produced a plan in a mean (± SD) time of 16 (± 6) min without any user intervention.

**TABLE 2:** Quantitative results of dose metrics for the original plans and the automatic field‐in‐field (FIF) plans related to the eyes, lenses, and brain structure

**Region of interest**	**Dose metric**	**Mean (± SD) original plan dose (Gy)**	**Mean (± SD) auto FIF dose (Gy)**
Left eye	Mean	14.95 ± 4.84	11.48 ± 2.03
Right eye		14.66 ± 5.50	11.44 ± 1.68
Left lens	Maximum	14.75 ± 9.13	8.19 ± 4.15
Right lens		15.77 ± 9.97	7.72 ± 2.96
Brain	D_1_	32.01 ± 0.35	32.21 ± 0.29
	D_95_	30.65 ± 0.23	30.63 ± 0.25
	D_99_	30.37 ± 0.26	30.33 ± 0.17

### 3.2 Qualitative algorithm evaluation

The results of the physician review of the FIF algorithm using the five‐point scale are listed in Table 3. In summary, 76.5% of the FIF plans were scored as clinically acceptable (needing no or minor stylistic edits) after renormalization of initially unacceptable plans. All of the plans were scored as clinically acceptable after minor clinically necessary edits, and no plans needed major edits.

**TABLE 3:** Results of the physician review of 17 automatically produced whole‐brain radiotherapy (WBRT) plans using a five‐point scale before and after renormalization

	Physician review scores (n)					Clinical acceptability (%)		
Normalization	5	4	3	2	1	Acceptable without edits	Acceptable with minor edits	Requires major edits
99.9%	5	7	3	2	0	70.6	88.0	11.8
Renormalized plans (99.4–99.7% of the Rx)	5	8	4	0	0	76.5	100.0	0

**FIGURE 5:**
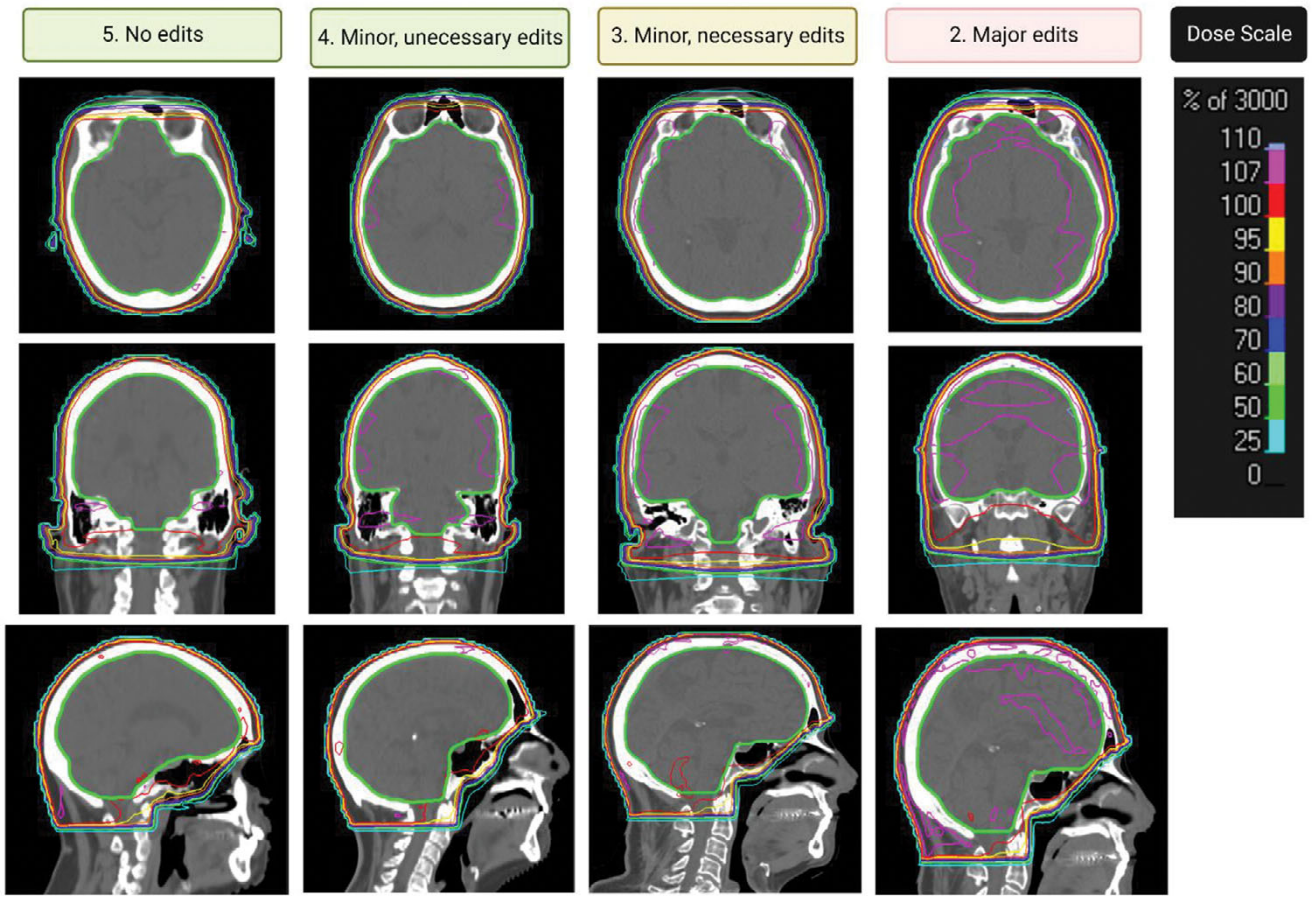
Examples of dose distributions in the sagittal, coronal, and axial views for the whole‐brain radiotherapy (WBRT) plans with scores of 2 or higher. The thickened green shape indicates the brain structure

Of the 17 plans reviewed by the physician, 29.4% were initially given a score of 3 or lower (needing minor or major edits). After renormalization of these plans, the overall clinical acceptability improved from 70.6% to 76.5%. The applied renormalization ranged from 99.4% to 99.7% of the Rx. In addition, the percentage of plans that were acceptable after minor clinically necessary edits improved from 88% to 100%, and the percentage of plans needing major edits decreased from 11.8% to 0%. Figure 5 shows the examples of dose distributions in the axial, coronal, and sagittal views for the plans with scores of 2 or higher assigned by the physician across the 17 patients. The reason for a plan to be scored as 3 was coldspots in the brain volume. The examples in Figure 5 with scores of 3 or higher were from plans after renormalization. After renormalization, none of the plans was scored 2 or below. The example plan in Figure 5 with a score of 2 was from prerenormalization.

## 4 DISCUSSION

In this work, we developed, validated, and clinically reviewed an end‐to‐end FIF algorithm that automatically produces treatment plans for WBRT. The results of the physician review and quantitative dose study showed that the FIF optimization algorithm can consistently create clinically acceptable plans for most patients. All of the parameters discussed for the FIF algorithm can be configured for adaptation to other clinical practices and physician preferences. In addition to the WBRT planning examined in this study, the algorithm has been previously tested and validated for rectal and cervical cancer treatment with three‐field and four‐field geometries, respectively.^18^, ^20^ Our study showed that the FIF algorithm can be extended to a two‐field geometry and whole brain, which is a different disease site.

The automated FIF algorithm has the potential to reduce manual planning time and enable clinics to devote limited resources to other tasks. The algorithm was developed with the goal of reducing the WBRT planning workload for staff of resource‐constrained clinics. This end‐to‐end automation process can greatly increase efficiency in the WBRT planning workflow and allow dosimetrists to focus resources on other clinical tasks. This algorithm may also decrease the barriers to implementation for resource‐constrained clinics in adopting the FIF technique in their clinical workflow without increasing their burdens.

The FIF algorithm uses a previously validated tool to generate field apertures based on anatomical landmarks.^12^ This landmark‐based field aperture generation approach can be customized to address various clinical and patient‐specific needs. Compared to the deep learning‐based approaches^11^, ^13^, the landmark‐based approach does not require additional training or model finetuning to customize or generate new field apertures. Combined with the landmark‐based aperture generation, the automated FIF planning can accommodate various planning requirements and calculate dose interactively without resources to train additional models.

To the best of our knowledge, this is the first automated treatment planning tool designed for WBRT with the FIF technique, which uses two opposed lateral beam to irradiate the brain structure and subfields to reduce hotspots and achieve a more homogeneous dose distribution. Specifically, the algorithm reduces dose inhomogeneities by using subfields to progressively block out high‐dose regions while maintaining the prescribed dose to the brain structure. The exact normalization needed to achieve the dose coverage may differ for different patients in different clinical practices. In the present study, we used a fixed normalization level as an input to the FIF algorithm. We found that renormalization of automatically produced plans after use of the FIF algorithm readily reduced hotspots further and improved plan acceptance without the need for any time‐consuming changes. After renormalization, none of the auto‐plans needed major edits, and one of the initially unacceptable plans became clinically acceptable.

The FIF algorithm has only been tested in our clinical practice. Different clinics, guidelines, and physicians may have different objectives for hotspots and dose coverages. Deployment of the algorithm in any clinic would require rigorous examination of the resulting automatically produced plans with respect to the local clinical practices and standards. Future studies may evaluate the performance of FIF optimization and the clinical acceptability of plans based on other clinical guidelines and practices. Key considerations for clinical deployment of this automated treatment planning tool include ensuring that the patient selection, and CT scans are appropriate for the treatment technique. The integrated workflow should undergo extensive stress testing and regular performance checks to ensure safety and consistency.

## 5 CONCLUSION

The FIF algorithm successfully produced clinically acceptable WBRT plans and can improve treatment planning efficiency when incorporated into an automatic planning workflow.

## CONFLICT OF INTEREST

The authors declare that there is no conflict of interest that could be perceived as prejudicing the impartiality of the research reported.

## FUNDING INFORMATION

Wellcome Trust, Cancer Prevention and Research Institute of Texas (CPRIT), and Dr. John and Mrs. Charlene Kopchick training fellowship.

## AUTHOR CONTRIBUTIONS

All authors contributed substantially to the conception, data acquisition, analysis, writing, and editing of the manuscript.
